# Supplementary material for: Synthetic multiantigen MVA vaccine COH04S1 protects against SARS-CoV-2 in Syrian hamsters and non-human primates
Source: NPJ Vaccines. 2022 Jan 21;7:7. doi: 10.1038/s41541-022-00436-6 (PMC8782996; doi:10.1038/s41541-022-00436-6)
Supplement: Supplementary file 1 — Supplementary information [file 41541_2022_436_MOESM1_ESM.pdf]

Synthetic Multiantigen MVA Vaccine COH04S1 Protects Against SARS-CoV-2 in Syrian Hamsters and Non-Human Primates

Supplementary Information

Supplementary Table 1. SARS-CoV-2 VOC-specific S mutations in pseudovirus particles

VOC	CDC Classification	Substitutions/Deletions (Δ)
B.1.1.7	Alpha	Δ69/70, Δ144, N501Y, A570D, D614G, P681H, T716I, S982A, D1118H
B.1.351	Beta	L18F, D80A, D215G, Δ242-244, R246I, K417N, E484K, N501Y, D614G, A701V
P.1	Gamma	L18F, T20N, P26S, D138Y, R190S, K417T, E484K, N501Y, D614G, H655Y, T1027I, V1176F
B.1.617.2	Delta	T19R, G142D, Δ156-157, R158G, A222V, L452R, T478K, D614G, P681R, D950N

Supplementary Table 2. Scoring parameters used to evaluate hamster lung histopathology

Grade	Severity	Findings
1	Minimal	histopathologic change ranging from inconspicuous to barely noticeable but so minor, small, or infrequent as to warrant no more than the least assignable grade. For multifocal or diffusely-distributed lesions, this grade was used for processes where less than approximately 10% of the tissue in an average high-power field was involved. For focal or diffuse hyperplastic/hypoplastic/ atrophic lesions, this grade was used when the affected structure or tissue had undergone a less than approximately 10% increase or decrease in volume.
2	Mild	histopathologic change that is a noticeable but not a prominent feature of the tissue. For multifocal or diffusely-distributed lesions, this grade was used for processes where between approximately 10% and 25% of the tissue in an average high-power field was involved. For focal or diffuse hyperplastic/hypoplastic/atrophic lesions, this grade was used when the affected structure or tissue had undergone between an approximately 10% to 25% increase or decrease in volume.
3	Moderate	histopathologic change that is a prominent but not a dominant feature of the tissue. For multifocal or diffusely-distributed lesions, this grade was used for processes where between approximately 25% and 50% of the tissue in an average high-power field was involved. For focal or diffuse hyperplastic/hypoplastic/atrophic lesions, this grade was used when the affected structure or tissue had undergone between an approximately 25% to 50% increase or decrease in volume.
4	Marked	histopathologic change that is a dominant but not an overwhelming feature of the tissue. For multifocal or diffusely-distributed lesions, this grade was used for processes where between approximately 50% and 95% of the tissue in an average high-power field was involved. For focal or diffuse hyperplastic/hypoplastic/atrophic lesions, this grade was used when the affected structure or tissue had undergone between an approximately 50% to 95% increase or decrease in volume.
5	Severe	histopathologic change that is an overwhelming feature of the tissue. For multifocal or diffusely-distributed lesions, this grade was used for processes where greater than approximately 95% of the tissue in an average high-power field was involved. For focal or diffuse hyperplastic/hypoplastic/atrophic lesions, this grade was used when the affected structure or tissue had undergone a greater than approximately 95% increase of decrease in volume.

Supplementary Table 3. NHP vaccine groups and gross pathological findings

Group	Study	Weight (kg)	Sex	Necropsy		Gross pathology findings at Necropsy
				day 7	day 21	
Mock	2D	4.76	F		X	All lobes normal
		3.02	F	X		Right caudal lung, remaining lobes normal. Overall hemorrhaging
		3.78	F		X	Left and Right caudal lung, remaining lobes normal
	1D	3.56	F	X		Pathology and congestion in all lobes
		3.45	F		X	Pathology and congestion in all lobes
		6.79	M		X	Left and Right caudal lung, remaining lobes normal
sMVA	2D	4.03	F	X		Right caudal lung, remaining lobes normal
		3.31	F	X		Gross congestion, possibly due to BALs. Lobes normal
		3.63	F		X	Right caudal lung, remaining lobes normal
	1D	3.41	F	X		Gross congestion, possibly due to BALs. Lobes normal
		3.73	F	X		Gross congestion, possibly due to BALs. Lobes normal
		6.05	M		X	All lobes normal
COH04S1	2D	4.02	F	X		Gross congestion, possibly due to BALs. Lobes normal
		2.91	F	X		Right caudal lung, remaining lobes normal
		3.69	F		X	Gross congestion, possibly due to BALs. Lobes normal
		3.68	F		X	Right caudal lung, remaining lobes normal
		3.37	F	X		Right caudal lung, remaining lobes normal
		7.23	M		X	Right caudal lung, remaining lobes normal
	1D	3.79	F	X		Gross congestion, possibly due to BALs. Lobes normal
		3.83	F		X	Right caudal lung, remaining lobes normal
		2.95	F	X		Gross congestion, possibly due to BALs. Lobes normal
		3.86	F		X	Gross congestion, possibly due to BALs. Lobes normal
		3.36	F	X		Right caudal lung, remaining lobes normal
		5.63	M		X	All lobes normal

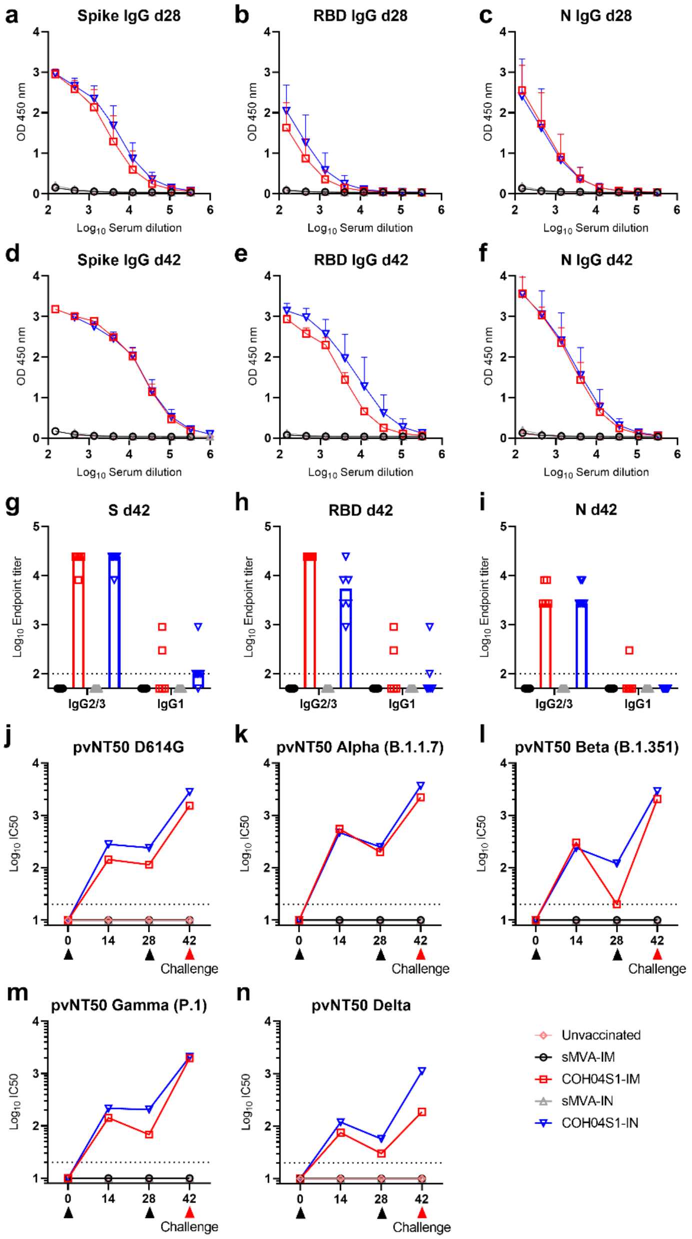

Supplementary Figure 1. Related to Figure 1. COH04S1 immunogenicity in hamsters. a-f. Binding antibody titers. Shown are binding curves of S, RBD, and N antigen-specific antibody titers measured at day 28 (d28) or day 42 (d42) after the first and second vaccination in serum samples of COH04S1-IM and COH04S1-IN-vaccinated hamsters and unvaccinated and sMVA vector control animals. Two-way ANOVA with Tukey's multiple comparison test was used **g-I. IgG2-3/IgG1 antibody titers.** Shown are IgG endpoint titers of S, RBD, and N antigen-specific IgG2-3 and IgG1 binding antibodies measured in serum samples of day 42 (d42). Bars indicate geometric means. Dotted lines indicate lower limit of detection. **j-n. VOC-specific NAb titers.** Shown are NAb titers measured in vaccine and control groups at the indicated time points by PV variants with D614G mutation or mutations based on several SARS-CoV-2 VOC. Dotted lines indicate lower limit of detection. Black triangles indicate time of vaccination. Red triangles indicate time of challenge.

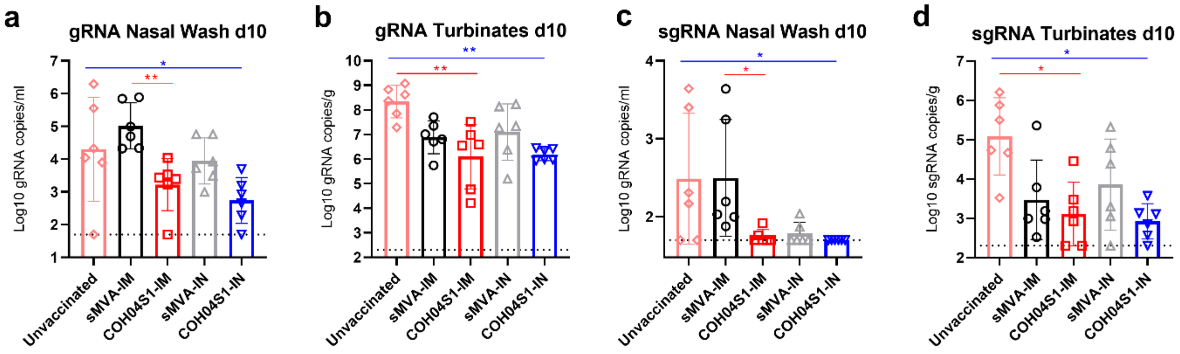

Supplementary Figure 2. Related to Figure 2. COH04S1-mediated vaccine protection of hamsters following sub-lethal SARS-CoV-2 challenge. Shown are SARS-CoV-2 genomic RNA (gRNA) and sub-genomic RNA (sgRNA) copies measured by qPCR in nasal wash and nasal turbinates of COH04S1-IM and COH04S1-IN-vaccinated hamsters and unvaccinated and sMVA control animals at day 10 post-challenge. Bars show RNA copies geometric mean ± geometric s.d.. Dotted lines represent lower limit of detection of the assay. Kruskal-Wallis test followed by Dunn's multiple comparison test was used. *=0.05 < p <0.01, **=0.01 < p < 0.001.

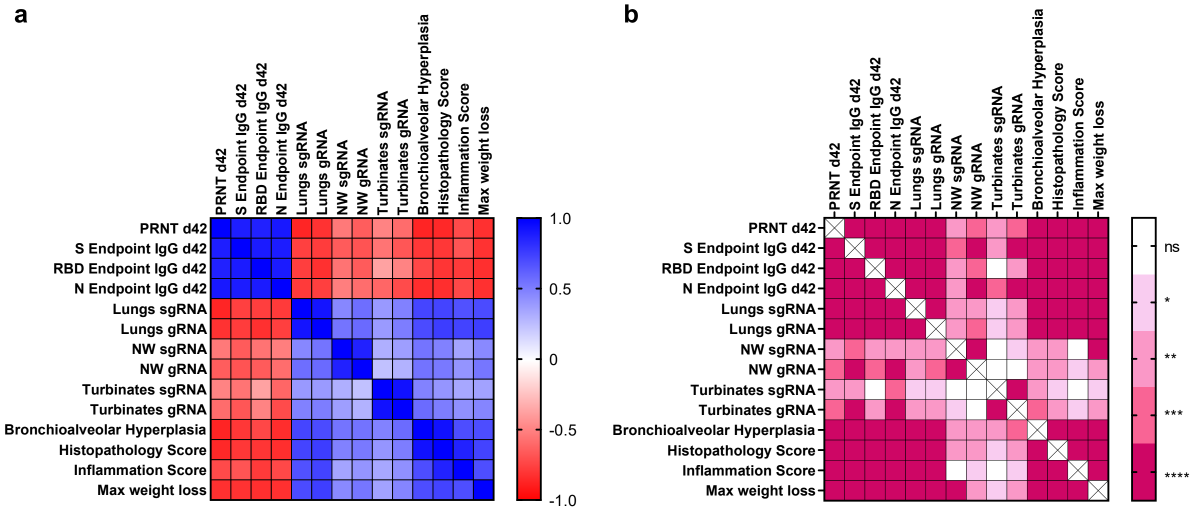

Supplementary Figure 3. Related to Figures 1 and 2. Correlative analysis of immunological and virological parameters in COH04S1-vaccinated hamsters challenged with SARS-CoV-2. A Spearman correlation analysis was performed between the indicated pre-challenge immune responses and post-challenge virological assessments of COH04S1-IM and COH04S1-IN-vaccinated hamsters and unvaccinated and sMVA vector control animals. **a.** Spearman correlation coefficients were calculated and plotted as a matrix. **b.** Two-tailed p values were calculated and indicated as: *=0.05 < p <0.01, **=0.01 < p < 0.001, ***=0.001 < p < 0.0001, ****=p < 0.0001. ns= not significant.

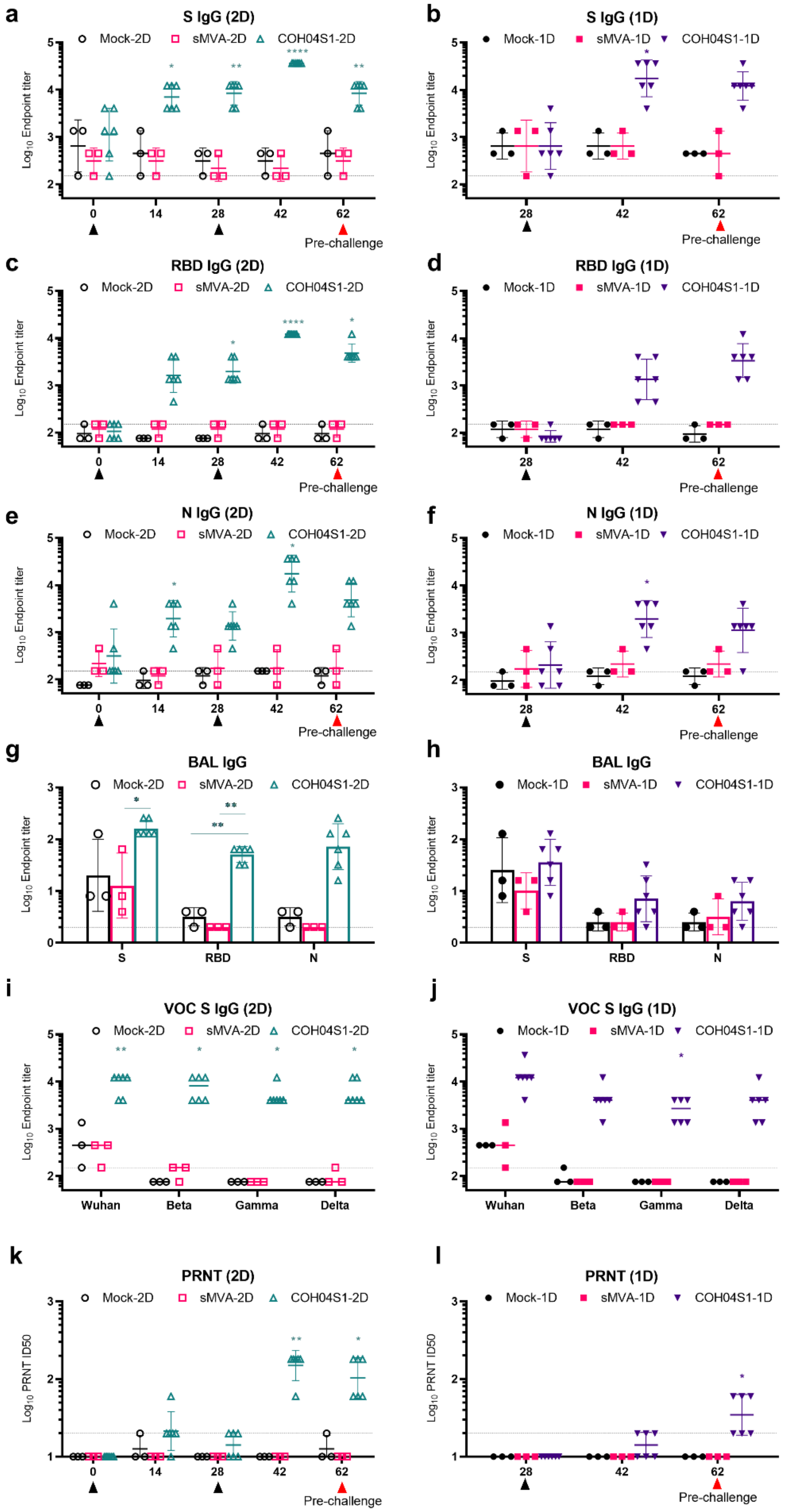

a
S IgG (2D)
Mock-2D sMVA-2D COH04S1-2D
Log10 Endpoint titer
0 14 28 42 62
Pre-challenge
b
S IgG (1D)
Mock-1D sMVA-1D COH04S1-1D
28 42 62
Pre-challenge
c
RBD IgG (2D)
d
RBD IgG (1D)
e
N IgG (2D)
f
N IgG (1D)
g
BAL IgG
S RBD N
h
BAL IgG
i
VOC S IgG (2D)
Wuhan Beta Gamma Delta
j
VOC S IgG (1D)
k
PRNT (2D)
Log10 PRNT ID50
l
PRNT (1D)

Supplementary Figure 4. Related to Figure 3. COH04S1-induced humoral immunity in NHP. a-f. Binding antibody titers. Shown are S, RBD, and N antigen-specific IgG endpoint titers measured at the indicated time points of the study (Figure 3a) in COH04S1-2D and COH04S1-1D-vaccinated NHP and mock-vaccinated and sMVA vector control animals. Data are presented as geometric mean values ± geometric s.d.. Two-way ANOVA followed by Sidak's multiple comparison test was used. **g-h. BAL binding antibodies**. Shown are endpoint titers of IgG binding antibodies to S, RBD, and N measured in bronchoalveolar lavage (BAL) samples in vaccine and control groups at day 42 of the study (Figure 3a) two weeks after 2D or 1D vaccination. Endpoint titers are presented as geometric mean values ± geometric s.d.. Two-way ANOVA with Sidak's multiple comparison test was used. **i, j. VOC-specific antibody titers**. Shown are endpoint titers of S-specific IgG binding antibodies measured at day 62 of the study (Figure 3a) by ELISA with S antigens based on Wuhan-Hu-1 reference strain or several VOC, including Beta (B.1.351), Gamma (P.1), and Delta (B.1.617.2). Endpoint titers are presented as geometric mean values ± geometric s.d.. Two-way ANOVA with Sidak's multiple comparison test was used. **k, l. PRNT NAb titers**. NAb titers were measured by PRNT assay against SARS-CoV-2 infectious virus in serum samples of vaccine and control groups. Serum dilutions reducing the plaque number by 50% (ID50) are presented as geometric mean values ± geometric s.d. Two-way ANOVA followed by Tukey's multiple comparison test was used to compare ID50 values. Black triangles indicate time of vaccination, red triangles indicate time pre-challenge sampling. Dotted lines represent assay lower limit of detection. $*=0.05 < p < 0.01$, $**=0.01 < p < 0.001$, $***=0.001 < p < 0.0001$, $****=p < 0.0001$.

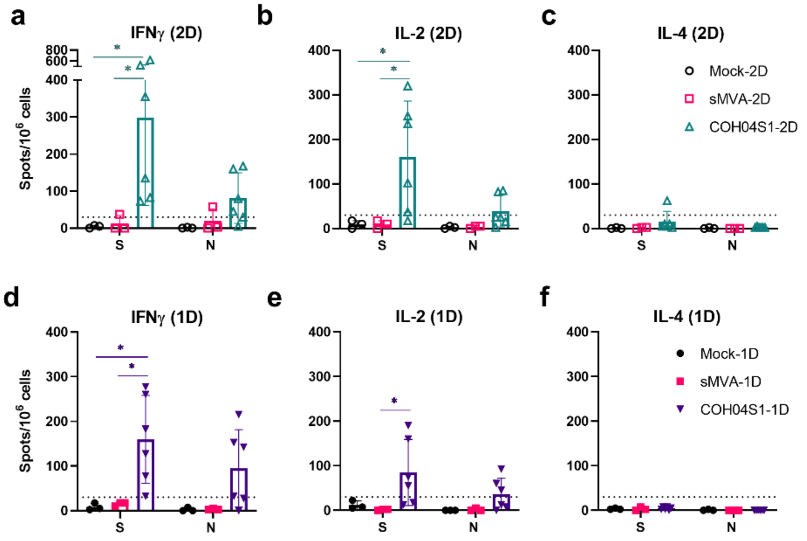

Supplementary Figure 5. Related to Figure 3. COH04S1-induced T cell responses in NHP. a-f. Shown are IFNγ, IL-2, and IL-4-expressing S- and N-specific T cell responses measured at day 42 of the study (Figure 3a) by Fluorospot assay in COH04S1-2D and COH04S1-1D-vaccinated NHP and mock-vaccinated and sMVA vector control animals. Bars represent mean values, lines represent ±s.d.. Two-way ANOVA followed by Tukey's multiple comparison test was used. Dotted lines indicate the arbitrary positive threshold of 30 spots/10^6 cells. *=0.05 < p <0.01.

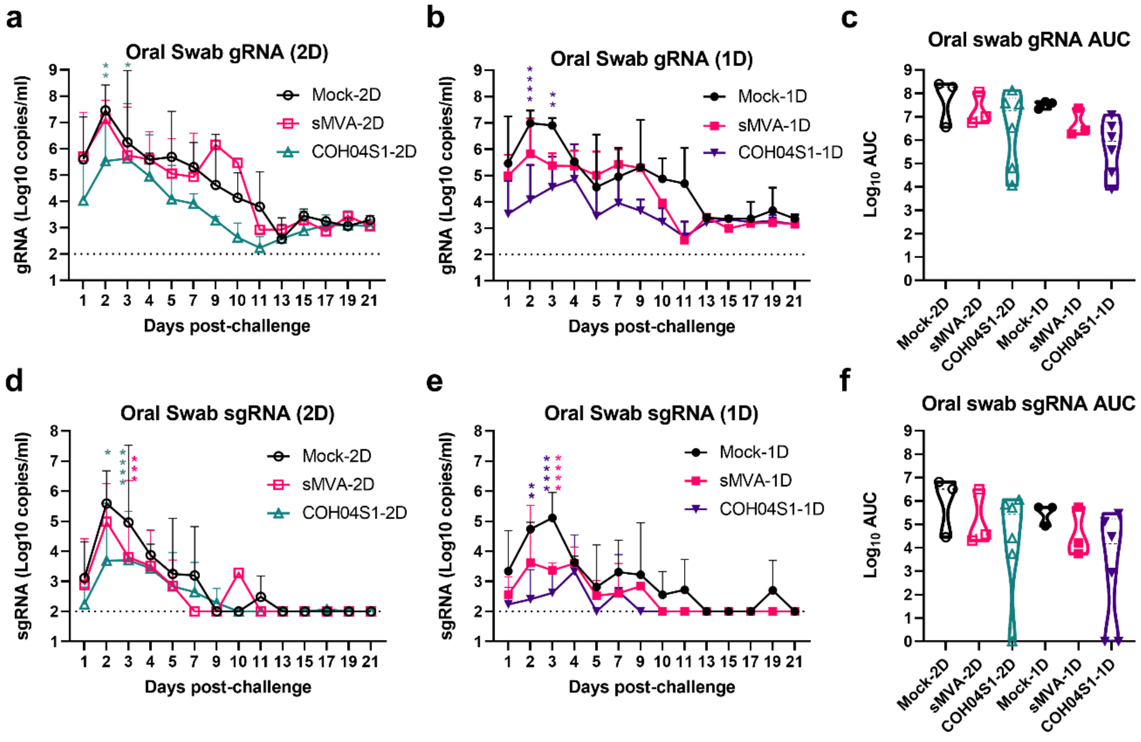

Supplementary Figure 6. Related to Figure 4 and 5. Oral swab viral loads in COH04S1-vaccinated NHP following SARS-CoV-2 challenge. SARS-CoV-2 genomic RNA (gRNA; **a-c**) and subgenomic RNA (sgRNA; **d-f**) copies were measured by qPCR at the indicated days post challenge in oral swab samples of COH04S1-2D and COH04S1-1D-vaccinated NHP and mock-vaccinated and sMVA vector control animals. Lines indicate geometric means ± geometric s.d.. Dotted lines represent assay lower limit of detection. Two-way ANOVA followed by Sidak's multiple comparison test was used. Panels in **c** and **f** show viral loads by area under the curve (AUC). Violin plots show values ranges with median values (dashed line) and quartiles (dotted line). AUC=0 was indicated as 1. One-way ANOVA followed by Tukey's multiple comparison test was used. *=0.05 < p <0.01, **=0.01 < p < 0.001, ***=0.001 < p < 0.0001, ****=p < 0.0001.

Figure S7

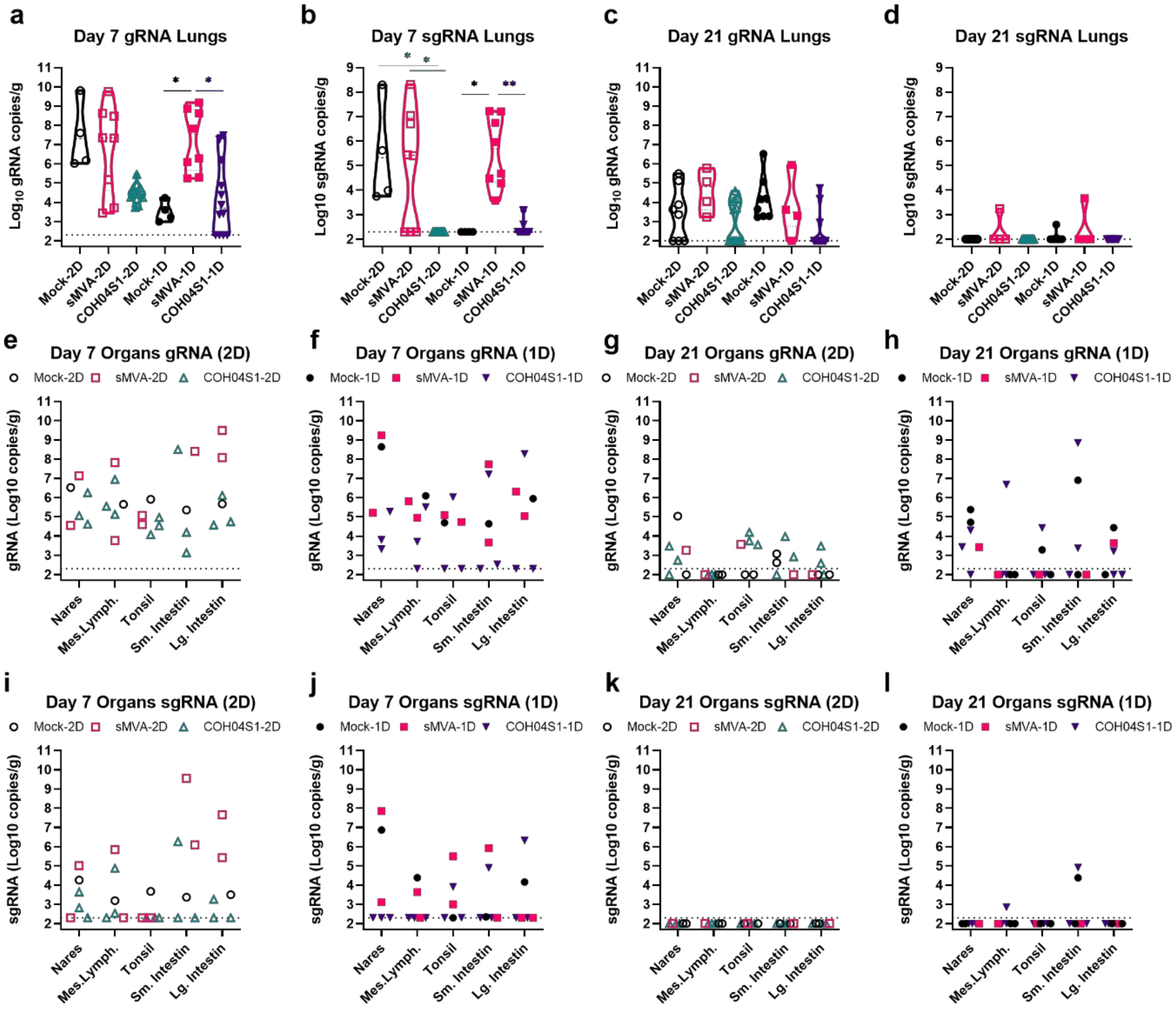

Supplementary Figure 7. Related to Figures 4 and 5. SARS-CoV-2 viral loads in lungs and other organs of COH04S1-vaccinated NHP following challenge. Given are SARS-CoV-2 genomic RNA (gRNA; **a, c, e-h**) and subgenomic RNA (sgRNA; **b, d, i-l**) copies measured by qPCR at day 7 and 21 post challenge in the lungs and several other organs of necropsied COH04S1-2D and COH04S1-1D-vaccinated NHP and mock-vaccinated and sMVA vector control animals. Four lung samples were analyzed for each necropsied NHP (see Supplementary Table 3 for necropsy schedule). Dotted lines represent assay lower limit of detection. Kruskal-Wallis test followed by Dunn's multiple comparison test was used. In e-l statistical evaluation was not performed because of the limited number of samples in the control groups. *=0.05 < p <0.01, **=0.01 < p < 0.001

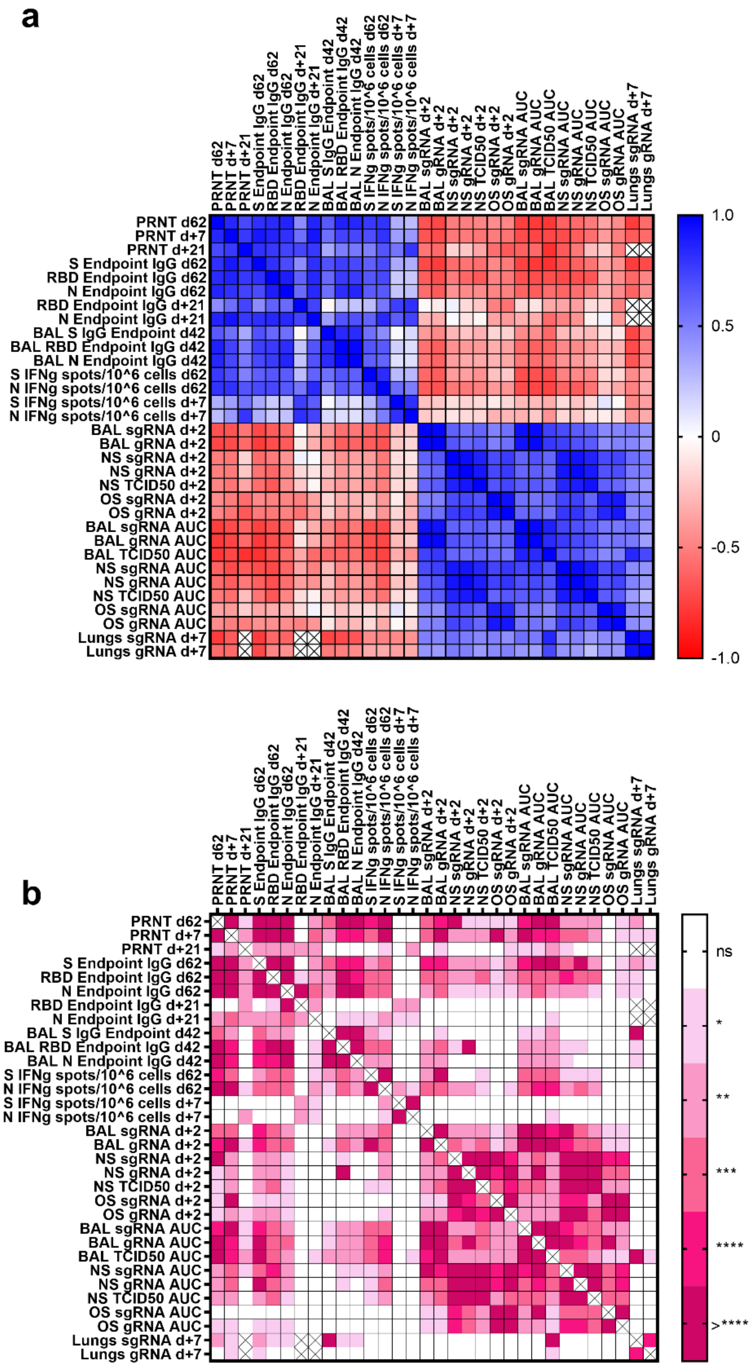

Supplementary Figure 8. Related to Figures 3 and 6. Correlative analysis of immunological and virological parameters in COH04S1-vaccinated NHP challenged with SARS-CoV-2. Spearman correlation analysis was performed between the indicated pre-challenge and post-challenge immune responses and post-challenge virological assessments of COH04S1-2D and COH04S1-1D-vaccinated NHP and mock-vaccinated and sMVA control animals. Post-challenge time-points are indicated with "+" before the day number. **a.** Spearman correlation coefficients were calculated and plotted as a matrix. **b.** Two-tailed p values were calculated and indicated as: *=0.05 < p <0.01, **=0.01 < p < 0.001, ***=0.001 < p < 0.0001, ****=p < 0.0001. ns= not significant.

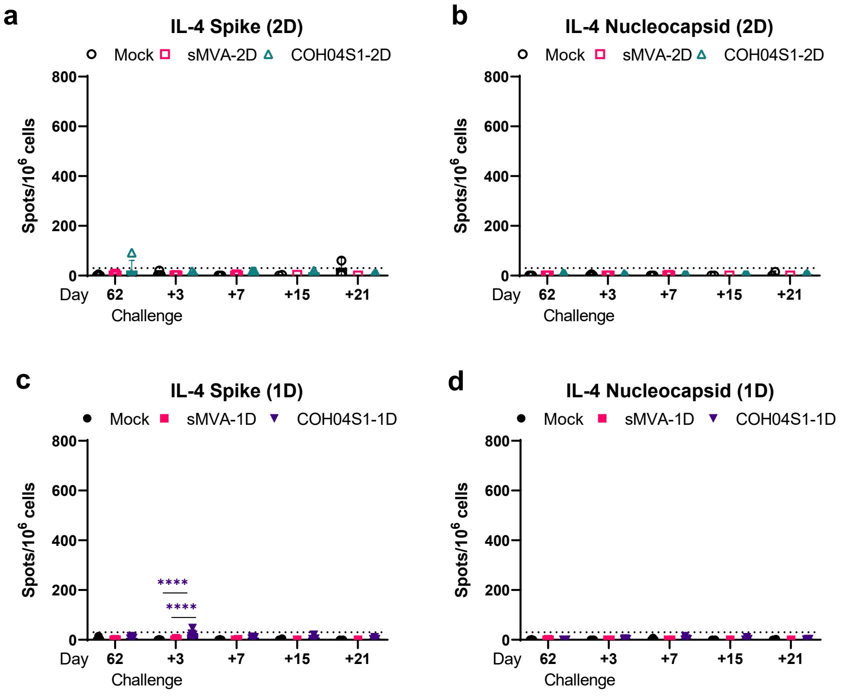

Supplementary Figure 9. Related to Figure 6. SARS-CoV-2-specific post-challenge cellular IL-4-specific responses in COH04S1-vaccinated NHP. SARS-CoV-2-specific cellular immune responses were measured at day 3, 7, 15 and 21 post-challenge in COH04S1-2D and COH04S1-1D-vaccinated NHP and mock-vaccinated and sMVA vector control animals and compared to pre-challenge immune responses assessed in these groups. IL-4-expressing S and N-specific T cell responses were measured by Fluorospot assay. Bars represent mean values, lines represent ± s.d.. Dotted lines indicate the arbitrary positive threshold of 30 spots/10^6 cells. Two-way ANOVA followed by Tukey's multiple comparison test was used. Time-points with <3 samples/group (d+15 and d+21) were excluded from the statistical evaluation. ****p < 0.0001.
