# Supplementary material for: Synthetic multiantigen MVA vaccine COH04S1 protects against SARS-CoV-2 in Syrian hamsters and non-human primates
Source: NPJ Vaccines. 2022 Jan 21;7:7. doi: 10.1038/s41541-022-00436-6 (PMC8782996; doi:10.1038/s41541-022-00436-6)
Supplement: Supplementary file 2 — REPORTING SUMMARY [file 41541_2022_436_MOESM2_ESM.pdf]

nature portfolio

Corresponding author(s): Felix Wussow

Last updated by author(s): Dec 7, 2021

Reporting Summary

Nature Portfolio wishes to improve the reproducibility of the work that we publish. This form provides structure for consistency and transparency in reporting. For further information on Nature Portfolio policies, see our Editorial Policies and the Editorial Policy Checklist.

Statistics

For all statistical analyses, confirm that the following items are present in the figure legend, table legend, main text, or Methods section.

n/a	Confirmed	
☐	☒	The exact sample size (n) for each experimental group/condition, given as a discrete number and unit of measurement
☐	☒	A statement on whether measurements were taken from distinct samples or whether the same sample was measured repeatedly
☐	☒	The statistical test(s) used AND whether they are one- or two-sided<br>*Only common tests should be described solely by name; describe more complex techniques in the Methods section.*
☒	☐	A description of all covariates tested
☐	☒	A description of any assumptions or corrections, such as tests of normality and adjustment for multiple comparisons
☐	☒	A full description of the statistical parameters including central tendency (e.g. means) or other basic estimates (e.g. regression coefficient) AND variation (e.g. standard deviation) or associated estimates of uncertainty (e.g. confidence intervals)
☐	☒	For null hypothesis testing, the test statistic (e.g. F, t, r) with confidence intervals, effect sizes, degrees of freedom and P value noted<br>*Give P values as exact values whenever suitable.*
☒	☐	For Bayesian analysis, information on the choice of priors and Markov chain Monte Carlo settings
☒	☐	For hierarchical and complex designs, identification of the appropriate level for tests and full reporting of outcomes
☐	☒	Estimates of effect sizes (e.g. Cohen's d, Pearson's r), indicating how they were calculated

Our web collection on statistics for biologists contains articles on many of the points above.

Software and code

Policy information about availability of computer code

Data collection	RT-qPCR data was collected with Applied Biosystems 7500<br>ELISPOT data was collected with CTL S6 Fluorocore (Immunospot)<br>Luminescence/Absorbance was collected with SoftMax Pro Software
Data analysis	RT-qPCR data was analyzed with Applied Biosystems 7500<br>ELISPOT data was analyzed with Fluoro-X™ FluoroSpot (Immunospot)<br>GraphPad Prism 8.3.0 and Microsoft Excel were used for data and statistical analyses

For manuscripts utilizing custom algorithms or software that are central to the research but not yet described in published literature, software must be made available to editors and reviewers. We strongly encourage code deposition in a community repository (e.g. GitHub). See the Nature Portfolio guidelines for submitting code & software for further information.

Data

Policy information about availability of data

All manuscripts must include a data availability statement. This statement should provide the following information, where applicable:

- Accession codes, unique identifiers, or web links for publicly available datasets
- A description of any restrictions on data availability
- For clinical datasets or third party data, please ensure that the statement adheres to our policy

All relevant data of this study are available from the corresponding authors upon request.

March 2021

Field-specific reporting

Please select the one below that is the best fit for your research. If you are not sure, read the appropriate sections before making your selection.

☒ Life sciences ☐ Behavioural & social sciences ☐ Ecological, evolutionary & environmental sciences

For a reference copy of the document with all sections, see nature.com/documents/nr-reporting-summary-flat.pdf

Life sciences study design

All studies must disclose on these points even when the disclosure is negative.

Sample size
Sample sizes included (a) n=6 hamsters/group and (b) n=6 agent-vaccinated NHP, n=3 empty vector-vaccinated NHP and n=3 mock-vaccinated NHP. There is not sufficient information about protective efficacy with these vaccine constructs to conduct a formal power analysis. For the hamster study, n=6 was sufficient to evaluate COH04S1 immunogenicity in a small animal model, based on previous studies in mice using the same vaccine (Chiuppesi 2020). For the AGM study, the group size of 3 and 6 was determined based on similar studies conducted by others with RSV (Le Nouen 2014; Cheng 2001; Taylor 2017). This number of animals represents the minimal number of animals necessary to evaluate vaccine replication relative to RSV and vaccine efficacy following SARS-CoV-2 challenge. An N of 3 AGM per group was used by Woolsey et al. (2020) to establish the suitability of the model for SARS-CoV-2 infection.

Data exclusions
No data were excluded. Day 5 nasal swab sgRNA and gRNA copies in COH04S1 vaccinated AGM (Fig. 5d-e) were not available at the time of submission.

Replication
Viral loads, pseudovirus neutralization were tested in triplicates. ELISPOT, ELISA and PRNT were tested in duplicates. Technical replicates presented minimal differences and were averaged.

Randomization
Sex was balanced when possible. Otherwise animals were randomly assigned to the groups.

Blinding
Animals were assigned a number and assays were performed with blinding when possible. In some assays, blinding to investigator and operators was not possible given the involvement in both sample preparation and analysis. Histopathology analysis was performed by a blinded (hamsters) or semi-blinded (NHP, mock-vaccinated animals were unblinded) board-certified Pathologist.

Reporting for specific materials, systems and methods

We require information from authors about some types of materials, experimental systems and methods used in many studies. Here, indicate whether each material, system or method listed is relevant to your study. If you are not sure if a list item applies to your research, read the appropriate section before selecting a response.

Materials & experimental systems

n/a	Involved in the study	
☐	☒	Antibodies
☐	☒	Eukaryotic cell lines
☒	☐	Palaeontology and archaeology
☐	☒	Animals and other organisms
☐	☒	Human research participants
☒	☐	Clinical data
☒	☐	Dual use research of concern

Methods

n/a	Involved in the study	
☒	☐	ChIP-seq
☒	☐	Flow cytometry
☒	☐	MRI-based neuroimaging

Antibodies

Antibodies used
anti-Hamster IgG(H+L) IgG3 (Southern Biotech 6061-05), anti-Hamster IgG1 IgG3 (Southern Biotech 1940-05), anti-HamsterIgG2/IgG3 IgG3 (Southern Biotech 1935-05), Goat anti-Monkey IgG (H+L) s (Thermo Fisher PA1-84631)

Validation
Southern Biotech 6061-05, 1940-05 and 1935-05 were validated by the vendor by ELISA.

Eukaryotic cell lines

Policy information about cell lines

Cell line source(s)
Vero E6 cells (ATCC, CRL-1586), HEK293T/17 cells (ATCC CRL11268), HEK293T-ACE2 cells (J.D.Bloom lab), Vero TMPRSS2 cells (Vaccine Research Center-NIAID), Vero E6-hACE2 cells (BEI Resources NR-53726)

Authentication
None of the cell lines were validated in-house. ACE2 expression on ACE2+ cells was periodically verified by FACS.

Mycoplasma contamination	Cell lines were not tested for Mycoplasma contamination in house.
Commonly misidentified lines (See ICLAC register)	No commonly misidentified lines were used in the study.

Animals and other organisms

Policy information about studies involving animals; ARRIVE guidelines recommended for reporting animal research

Laboratory animals	15 male and 15 female Syrian golden hamsters (Envigo), 6-8 weeks old. 4 male and 20 female research naive, adult, African green monkeys, 3-7Kg purchased from a Bioqual's approved vendor.
Wild animals	No wild animals were used in the study
Field-collected samples	The study did not involve field-collected samples
Ethics oversight	The study was approved by: Bioqual (protocols 20-163 and 20-120) and and City of Hope (protocols 20087 and 20075) Institutional Animal Care and Use Committees (IACUC)

Note that full information on the approval of the study protocol must also be provided in the manuscript.

Human research participants

Policy information about studies involving human research participants

Population characteristics	Male and female healthcare workers >18 years of age were enrolled in a observational study to evaluate humoral and cellular immunity to COVID-19 EUA vaccines
Recruitment	Volunteers signed informed consent
Ethics oversight	Protocol 20720 was approved by by the City of Hope (COH) Institutional Review Board (IRB)

Note that full information on the approval of the study protocol must also be provided in the manuscript.
